# From Simulation to Application: Droplet-Based Microfluidics for Thermal Targeting of Cancer Cells

**DOI:** 10.3390/mi17070782

**Published:** 2026-06-27

**Authors:** Zsombor Szomor, Eszter L. Tóth, János M. Bozorádi, Tamás Pardy, Rauno Jõemaa, Péter Fürjes

**Affiliations:** 1 Microsystems Laboratory, Institute of Technical Physics and Materials Science, HUN-REN Centre for Energy Research, 1121 Budapest, Hungary; szomor.zsombor@ek.hun-ren.hu (Z.S.); leelossyne.toth.eszter@ek.hun-ren.hu (E.L.T.); bozoradi.janos@ek.hun-ren.hu (J.M.B.); 2 Doctoral School on Materials Sciences and Technologies, Óbuda University, 1034 Budapest, Hungary; 3 Thomas Johann Seebeck Department of Electronics, Tallinn University of Technology, 19086 Tallinn, Estonia; tamas.pardy@taltech.ee (T.P.); rauno.joemaa@taltech.ee (R.J.)

**Keywords:** microfluidics, droplet generation, finite element modelling (FEM), mixing, heat shock, cell manipulation, thermal treatment

## Abstract

This paper presents the development, fabrication, and characterization of a droplet-based microfluidic platform designed for precise local thermal treatment of cancer cells, with prospective chemical targeting as a future application. The workflow begins with a finite element model (FEM) using COMSOL Multiphysics 6.0 to characterize coupled hydrodynamic and thermal behavior, specifically analyzing temperature distributions across single-phase and three-phase regimes. Following the simulation, work has progressed to the fabrication of a microfluidic device and the characterization of its platinum heat source and temperature detector to ensure precise thermal control. To replicate realistic biochemical conditions, experiments have employed a three-phase configuration of oil, water, and fluorescent BSA solution. In the final stage, DX5-GFP MES-SA cancer cells have replaced the BSA solution to complete the measurements. To ensure reagent homogenization and consistent cellular exposure, a serpentine channel design was utilized to induce Dean vortices, which significantly enhanced internal mixing within the droplets. Fluorescence-loss experiments demonstrated that localized heating above ~60 °C induces irreversible thermal damage in both model proteins (fluorescent BSA) and cancer cells, establishing a proof-of-concept basis for precise thermal regulation at the single-droplet level. By deactivating specific thermo-sensitive proteins responsible for drug resistance, this integrated approach to thermal and hydrodynamic optimization enhances the efficacy of chemical stimuli and provides a robust platform for investigating the modulation of cellular defense mechanisms in future biotechnological applications. The platform holds significant potential for advancing precision oncology by enabling systematic, single-cell-level investigation of heat-shock-mediated drug sensitization, with long-term implications for overcoming multidrug resistance in aggressive cancer therapies.

## 1. Introduction

Microfluidics has transformed biological and chemical analyses by enabling high-throughput and precise experimentation on miniaturized platforms [1]. Lab-on-a-chip systems facilitate tightly controlled reactions in microscale chambers, while reducing reagent consumption and assay time. The key requirements for these microreactors include efficient reagent mixing and accurate temperature regulation under laminar flow and microscale heat-transport conditions [2]. Thermal management is particularly critical in biochemical applications, such as polymerase chain reaction (PCR) and protein synthesis, where precise temperature control directly influences amplification efficiency, stability, and functionality [3,4,5,6,7,8,9,10,11]. Beyond synthesis, precise thermal control has been proposed as a potential tool for modulating the defense mechanisms of cancer cells against chemical agents. Heat-sensitive proteins, such as heat shock proteins (HSP) HSP70 and HSP90, act as molecular chaperones that maintain cellular proteostasis and enable survival under the stress of chemotherapy [12,13,14,15]. It has been hypothesized that by applying elevated localized temperatures, thermosensitive proteins may be disrupted, potentially lowering cellular resistance to subsequent chemical drug stimuli. However, it must be noted that the temperatures required for direct thermal killing (above ~55–60 °C) are substantially higher than the canonical heat-shock range (~41–43 °C) at which HSP upregulation is typically observed, and rigorous biological validation of HSP-mediated sensitization remains an important open question. The present work focuses on demonstrating the engineering platform for controlled localized heating; biological validation is identified as a key direction for future research.

The development of such platforms builds upon extensive prior research in droplet-based microfluidics and thermal control. Previous studies have demonstrated various methods for droplet generation and manipulation, emphasizing the advantages of compartmentalization for single-cell analysis [1]. Regarding thermal regulation, researchers like Hamad et al. [16] have utilized computational fluid dynamics (CFD) to optimize PCR thermal flow systems, while others have explored integrated microheaters for localized heating [17]. Building on these techniques, Prakash et al. [18] demonstrated the integration of droplet-based nucleic acid amplification with real-time detection, where precise thermal control is fundamental to achieving high sensitivity in viral diagnostics. Furthermore, Zhang and Jiang [19] highlighted that in continuous-flow droplet PCR, several coupled parameters must be carefully investigated, such as droplet residence time within specific thermal zones, the stability of thermal cycling, and the mitigation of cross-contamination. Beyond thermal aspects, efficient intradroplet mixing is equally vital for single-cell experiments to ensure reagent homogeneity and fast reaction kinetics. Yang et al. [20] demonstrated that rapid droplet-based mixing is essential for single-molecule spectroscopy and the study of surface-adhesive proteins, where traditional laminar flow would otherwise limit the analysis. Internal mixing within droplets was highlighted by the implementation of passive mixing structures, such as serpentine channels, to overcome diffusion limits in laminar regimes [21]. While these works have established the foundation for microscale thermal and mass transport, the integration of precise local heat shock treatment with high-efficiency intradroplet mixing specifically for targeting cancer cell defense mechanisms remains a complex challenge that requires further optimization [11]. Several prior engineering approaches have sought to study or exploit cancer-cell defense mechanisms at the microscale. Bulk hyperthermia chambers and water-bath systems have been used to apply thermal stress to cell populations, but these lack the spatial resolution needed to target individual cells or small clusters [12,15]. Microfluidic platforms employing resistive heaters have been reported for on-chip hyperthermia, yet most operate in continuous flow single-phase configurations that do not isolate individual cells from the bulk fluid environment. More recently, droplet-based encapsulation has been recognized as a route toward single-cell thermal assays, though integration with active on-chip heating and simultaneous mixing enhancement remains rare [1,21].

In this paper, we address these challenges by developing and characterizing a droplet-based microfluidic platform designed for controlled local thermal treatment of encapsulated cells, with the longer-term goal of combined thermal and chemical stimulation. The combination of droplet isolation, integrated Pt microheaters, and Dean vortex-driven mixing reported here represents a step toward a more controllable and reproducible platform for probing heat-shock-mediated cellular responses. The primary objective is to achieve precise local thermal control and uniform reagent homogenization within moving droplets to ensure consistent cellular exposure. In this context, precision refers to the ability to reproducibly deliver defined heat doses to individual droplets within a narrow and well-characterised temperature window, which has been demonstrated across a range of integrated platinum microheater platforms in the literature [3,16,17,22,23]. In the present study, this is supported by the near-linear resistance–temperature response of the integrated Pt heater and RTD elements closely matching the nominal platinum value, enabling real-time temperature estimation from resistance readings. Also, the spatial confinement of heating was confirmed by FEM simulations showing that peak temperatures above 60 °C remain localised within approximately 200 µm of the heater surface, while the repeatability of the thermal effect was demonstrated by consistent fluorescence-loss thresholds observed across independent droplets in the 60–65 °C range.

We present a comprehensive workflow starting with a 3D finite element model (FEM) to predict coupled hydrodynamic and thermal behavior across three-phase regimes (oil, water, and BSA/cells). This is followed by the fabrication and experimental validation of the device, featuring integrated platinum microheaters and a serpentine channel design to induce Dean vortices for enhanced mixing. Fluorescence-loss experiments using fluorescently labelled BSA (by Fluorescein isothiocyanate—FITC) and DX5-GFP MES-SA cancer cells are used to characterize the thermal thresholds for irreversible cellular damage at the single-droplet level. The MES-SA cell line is derived from uterine sarcoma and is widely employed as an in vitro model for investigating chemotherapeutic response and multidrug resistance mechanisms [24], making it a relevant model for future drug-combination studies on this platform.

What distinguishes the present approach from conventional bulk hyperthermia is the combination of droplet encapsulation, integrated on-chip heating, and active mixing within a single device. Confining cells at the single-droplet level allows spatially resolved thermal stimulation while minimizing reagent consumption and cross-contamination between replicates. Platinum microheaters and temperature detectors positioned directly beneath the channel deliver precise, rapidly adjustable heat doses that would be difficult to achieve with uniform bath heating. The serpentine channel geometry further ensures homogeneous distribution of chemical stimuli within each droplet through Dean vortex-driven mixing, which is essential for reproducible cell–reagent interactions. Supporting the experimental workflow, the multimodal FEM framework allows heater placement, flow rates, and thermal boundary conditions to be optimized computationally before fabrication. Together, these features provide a scalable foundation for controlled investigations of heat-shock-mediated modulation of cancer cell drug resistance and future combined thermal–chemical therapy studies. It should be noted that the current study constitutes an engineering proof-of-concept; biochemical validation of HSP-specific deactivation and drug-sensitization effects is beyond the scope of the present work and is planned as a dedicated future study.

## 2. Materials and Methods

### 2.1. Multimodal Modeling Framework for Droplet-Based Thermal and Mass Transport

Microfluidic architectures are typically categorized into chamber-based and continuous-flow designs, each of which offers unique advantages for thermal management. Droplet-based platforms—a specialized form of continuous flow—enable high-throughput parallel reactions and single-cell analysis. Maintaining precise temperature regulation, however, and efficient mixing at the droplet level remain a critical challenge. To optimize these systems, finite-element modeling (FEM) provides essential predictive insights into the complex coupling of fluid dynamics and heat transfer [17]. In this study, FEM was applied to a three-phase droplet-based system to evaluate the temperature distributions within locally heated droplets across varying flow rates and fluid compositions, specifically water, oil, and a fluorescent BSA solution. This modeling approach also allows for a detailed analysis of the mixing efficiency and reagent concentration distributions [21]. The three-phase configuration was optimized to balance biological compatibility, stable droplet generation, and thermal transport. The silicone oil served as the continuous carrier phase due to its biocompatibility and optical transparency; its low thermal conductivity acts as an insulation barrier that promotes heat retention within the droplets. Distilled water was utilized as the primary dispersed stream to replicate the aqueous intracellular environment, and a fluorescent BSA solution was co-injected as a secondary macromolecular protein surrogate at a concentration optimized for linear optical detection.

Building upon these principles, the current study has established a multiphysics numerical framework to analyze how microchannel geometry, material parameters, flow rates, and initial thermal states influence transient heat and mass transport. Implementation has utilized the finite element method (FEM) in COMSOL Multiphysics, which has enabled full three-dimensional simulations of droplet generation and transport within a three-phase configuration [25]. The numerical approach allows direct coupling between the hydrodynamic behaviour and species transport, thereby capturing the interplay between the flow structure and concentration evolution inside the moving droplets. In the simulated configuration, distilled water and fluorescently labelled bovine serum albumin (BSA) were introduced as parallel dispersed streams and segmented into quasi-monodisperse droplets using a continuous silicone oil phase. The fluid motion in all phases was described by the incompressible Navier–Stokes (1) equation, which expresses momentum conservation in the presence of viscous stresses and body forces [26]:

(1) ρ(∂u/∂t + u⋅∇u) = −∇p + ∇⋅[μ(∇u + (∇u)^T^ − ⅔μ(∇⋅u)I)] + F

While mass conservation was enforced through the continuity Equation (2):

(2) ∂ρ/∂t + ∇⋅(ρu) = 0

Here, ρ denotes the fluid density, u is the velocity vector, p represents pressure, and μ is the dynamic viscosity. The term F accounts for volumetric body forces arising primarily from interfacial effects in multiphase flow.

In the ternary phase-field formulation [21], the local fluid properties were evaluated as phase-averaged quantities that varied smoothly across diffuse interfaces. The effective viscosity and density were therefore defined as the weighted sums of the individual phase properties according to the local phase-field variables ϕ_i_, as given in (3) and (4):

(3) μ(ϕ) = Σ_i_ μ_i_ϕ_i_

(4) ρ(ϕ) = Σ_i_ ρ_i_ϕ_i_

where ϕ_i_ represents the local phase-field variable corresponding to phase i satisfying Σ_i_ϕ_i_ = 1.

To analyze droplet formation and stability, the influence of inlet flow rate ratio between dispersed and continuous phases was examined through systematic parametric sweep simulations. Interface dynamics between the immiscible fluids, silicone oil, water, and fluorescently labeled BSA solution, were captured using the ternary phase-field approach. This method explicitly accounts for interfacial tension and wetting behavior, and can be regarded as a generalization of the conventional Level Set [27,28] and binary phase-field models [29] to three-phase systems. In this formulation, fluid interfaces are represented as diffuse transition regions characterized by a finite thickness ε, which was selected based on the mesh resolution and characteristic geometric length scales to balance numerical stability with physical accuracy. The temporal evolution of the interfaces is governed by the mobility parameter M_0_, which controls the rate of phase redistribution while preserving interfacial integrity.

The ternary system is described by three phase-field variables ϕ_A_, ϕ_B_, and ϕ_C_, which correspond to phases A, B, and C, respectively. These variables are constrained such that their sum remains unity throughout the domain, ensuring the conservation of the phase volume fractions. The interfacial regions are identified by the ϕ_i_ = 0.5 isosurface, whereas surface tension effects arise naturally from gradients in the phase-field variables through the system’s free-energy functional. The evolution of phase fields is governed by a set of coupled Cahn–Hilliard equations [26]:

(5) ∂ϕ_i_/∂t = M∇^2^(∂Ψ/∂ϕ_i_ − Σ_j_ ≠_i_ γ_ji_∇^2^ϕ_j_)

In (5), ϕ_i_ denotes the phase-field associated with phase i, M is the mobility coefficient, Ψ represents the free-energy density of the ternary system, and γ_j__i_ is the interfacial tension coefficient between phases j and i. The Laplacian operator ∇^2^ accounts for the interfacial diffusion and curvature effects. These equations are fully coupled to the Navier–Stokes equations in (1), enabling the simultaneous resolution of the interfacial dynamics and fluid motion in three-phase microflows.

The body-force term (6) F_st_ accounts for the capillary contributions originating from the interfacial tension between the immiscible phases [30]. Within the ternary phase-field framework, these forces arise from the spatial gradients of the phase-field variables and are expressed as volumetric force density acting on the fluid:

(6) F_st_ = Σ_i_ η_i_∇ϕ_i_

where ϕ_i_ denotes the phase-field variable associated with phase i, and η_i_ represents the corresponding chemical potential derived from the free-energy functional. This formulation enables a direct and consistent coupling between interfacial dynamics and hydrodynamic flow by introducing surface-tension effects as source terms in the Navier–Stokes equations.

At the length scales considered in this study, additional body forces such as gravity, magnetic fields, and thermally induced forces were neglected because of their minimal influence on flow behavior. Fluid–solid interactions were modeled using prescribed static contact angles θ_i_, which define the wetting properties of the channel walls and suppress droplet adhesion. A contact angle of 90° was applied throughout the computational domain. Phase-dependent material properties, including density and viscosity, were smoothly varied across diffuse interfaces, which were identified by the ϕ_i_ = 0.5 isosurface.

To resolve the concentration fields within moving droplets, mass transport was modeled using the Transport of Diluted Species formulation, coupled to both the laminar flow and ternary phase-field interfaces. This approach captures advective and diffusive transport processes in dissolved phases while accounting for the evolving droplet geometry. The model tracks the molar concentration C_i_ of each species, including water, silicone oil, and BSA, according to the governing transport equation [31]:

(7) ∂(θC_i_)/∂t = ∇⋅(D_eff_,_i_∇C_i_) − v⋅∇C_i_ + R_i_

In (7), θ denotes the saturation, v is the local convective velocity obtained from the flow solution, C_i_ represents the molar concentration of species i, and D_eff,i_ is the effective diffusion coefficient. The reaction term R_i_ was set to zero in all simulations, as no chemical reactions were considered. For moving multiphase systems, the species transport equations are inherently coupled to the fluid velocity field, allowing convection-driven redistribution of solutes within the droplets.

The analysis has evaluated the thermal response of the microfluidic system by solving energy balance equations through a coupled heat transfer formulation [32]. This approach has enabled the prediction of temperature fields across media with varying thermal properties and has accommodated fluctuations in fluid composition. The model has accounted for both conductive and convective mechanisms, where local fluid velocities have driven thermal advection. Furthermore, the framework has coupled the thermal field to the hydrodynamic model, allowing the simultaneous resolution of fluid motion and heat transport. In addition, the formulation supports conjugate heat transfer, enabling the inclusion of solid domains, such as the substrate and integrated heating elements, within the same computational framework. The governing Equations (8) and (9) for transient heat transfer in moving fluids are given by:

(8) ρC_p_(∂T/∂t) + ρC_p_u⋅∇T + ∇⋅q = Q + Q_vd_ + Q_p_

(9) q = −κ∇T

where ρ denotes the fluid density, C_p_ is the specific heat capacity at constant pressure, u is the flow velocity, T is the temperature, q is the heat flux, and κ is the thermal conductivity. The term Q corresponds to a general heat source, whereas Q_vd_ accounts for the heat generated by viscous dissipation owing to shear. An additional term, Q_p_, can be included to represent the heating from pressure work, which may become significant under adiabatic compression conditions.

The material parameters used in the numerical model were defined based on a combination of reference database values, manufacturer datasheets, and literature reports, as summarized in Table 1, Table 2 and Table 3. All properties were specified at a reference temperature of approximately 20–25 °C and were treated as temperature-independent constants throughout the simulations. The thermophysical properties of distilled water were obtained from standard reference data. The parameters for the silicone oil continuous phase were selected according to supplier specifications and published sources (Sigma Aldrich—CAS 63148-58-3, Damstadt, Germany) [33,34,35,36], while the properties of the fluorescent BSA solution were taken from previously reported experimental studies [17,21].

After defining the material properties in the numerical model, volumetric flow rates were prescribed at all inlet boundaries to establish continuous flow conditions within the microchannel, whereas a zero-pressure condition was applied at the outlet. To achieve seamless imaging during screening, the concentration of the BSA solution was specifically matched to the optical density of the target cell population (approximately 2.3 × 10^6^ cells/mL). The fluid was treated as incompressible throughout the simulations. The droplet generation and mixing section of the microfluidic device had a uniform height of 50 μm and an initial width of 200 μm, which expanded to 400 μm upstream of the 4.5 mm-long main channel. Two platinum microheaters, each with lateral dimensions of 600 × 400 μm^2^, were positioned beneath the channel, with the second heater located 1500 μm downstream of the first. By appropriately tuning the applied volumetric heating power for the different fluid phases (water, silicone oil, and BSA solution), the temperature at the surface of the second heater was adjusted to reach approximately 90 °C.

### 2.2. Microfluidic Chip Fabrication and Experimental Validation

**Figure 1:**
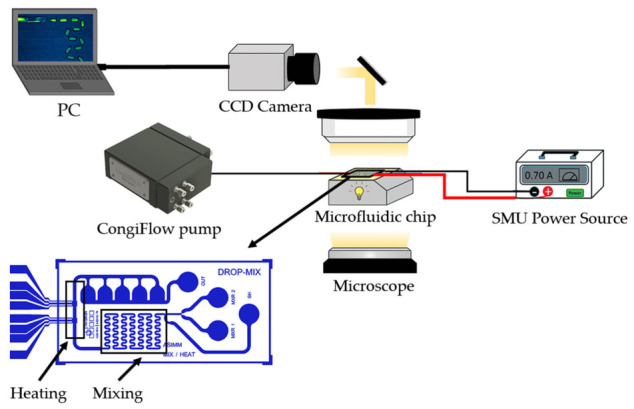
Measurement setup for the experimental validations.

**Figure 2:**
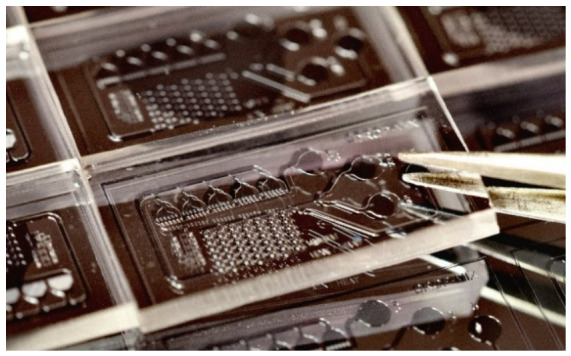
The fabricated microfluidic chip produced by soft-lithography using PDMS polymer.

Experimental measurements were conducted to validate the simulation results (Figure 1). The experimental work was carried out at the Microsystems Laboratory of the HUN-REN Centre for Energy Research, where basic microtechnological and micromechanical steps were implemented for processing microfluidic systems. Soft lithography, recognized as one of the most efficient techniques for processing microfluidic structures, was utilized to fabricate the structures in a polydimethylsiloxane (PDMS) polymer material through a casting method (Figure 2). The SU-8 master mold defined channels with a uniform height of 50 μm throughout the device. The inlet channels for all phases (water, BSA and oil) each had a width of 200 μm. These channels converged at a flow-focusing T-junction, after which the combined stream entered the droplet generation region with a channel width of 200 μm. Each serpentine mixing section consisted of 12 curved segments, each with a radius of curvature of 200 μm. After the serpentine-type mixing channel, the main channel widened to 400 μm to accommodate the formed droplets and reduce shear stress. This main heating channel was approximately 12 mm long and 400 μm wide, positioned directly above the two integrated Pt microheaters.

**Figure 3:**
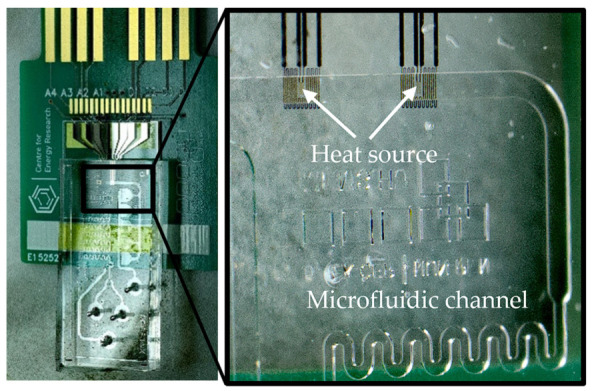
The fabricated heat sources and RTDs with the microfluidic system on a Borofloat glass substrate. The connection of the heat source was wire-bonded to a PCB for electrical control.

A platinum heat source was formed on a Borofloat^TM^ glass substrate, chosen for its mechanical stability and chemical inertness. Before deposition, the substrate was treated with 65% HNO_3_ at 90 °C and rinsed with 18 MΩ·cm deionized water to achieve a clean, organic-free surface. A 1.8 µm thick sacrificial photoresist was patterned prior to metal deposition to define the heater and detector geometry via lift-off. Thin-film deposition proceeded in two steps: a 10 nm Ti adhesion layer was first applied to ensure reliable adhesion to the glass, followed by a ~270 nm Pt layer deposited by magnetron sputtering (Vaksis). The lift-off step then dissolved the underlying resist, yielding precisely patterned Pt metal wires on the substrate surface. The heat source and the resistance temperature detector (RTD) fabricated with the microfluidic device is shown in Figure 3. A Keithley 2635A source-measure unit (SMU, produced by Farnell, Leeds, UK) was used to supply power and regulate the operation of the integrated platinum heating element. A microfluidic channel was bonded onto a Borofloat^TM^ glass substrate above the heater, enabling temperature characterization under different channel conditions.

A CogniFlow piezoelectric micropump was used to introduce liquid into the system [37]. The operation of the implemented microfluidic chips with an integrated heat source has been examined using dark-field and fluorescence microscopy. A Zeiss Axio Vert A1 inverted microscope was used for the measurements. In the case of the CogniFlow micropump, both pressure and frequency can be adjusted to achieve the appropriate conditions for droplet formation. Each liquid had a default frequency value that enabled efficient pumping. For the oil phase, the frequency was set to 30 Hz, while for the water and BSA solutions, it was set to 200 Hz, as these two liquids have similar viscosities and densities. After adjusting the frequencies, the pressure values of the individual liquid phases had been tuned while maintaining the previously defined pumping frequencies to generate droplets with sizes comparable to the channel width and to enable the observation of mixing inside the droplets after their formation. By adjusting the pressure difference between the continuous oil phase and aqueous phases (water and BSA solution), both the size and generation rate of the droplets could be regulated.

## 3. Results

### 3.1. Thermal Field Characterization in Continuous Single-Phase Microflows

**Figure 4:**
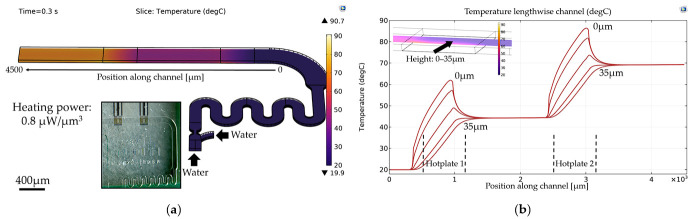
Simulated temperature distribution within the microfluidic channel for single-phase water flow at a total volumetric flow rate of 1.8 μL/s (**a**). Longitudinal temperature profiles extracted along the channel centerline under steady-state conditions are shown in (**b**). To quantify transverse heat distribution, five cut lines were defined at the channel mid-plane at vertical positions of 0, 5, 15, 25, and 35 μm. The integrated micro-heaters were also fabricated and independently characterized through experimental temperature measurements.

Solving the coupled flow and heat transfer equations yields the steady-state velocity and temperature fields shown in Figure 4. The color map illustrates the steady-state temperature field within the microfluidic channel for single-phase water flow, as shown in Figure 4a. Corresponding one-dimensional temperature profiles extracted along the channel length at different vertical positions relative to the heat source are shown in Figure 4b. For this analysis, the total volumetric flow rate was fixed at 1.8 μL/s, and identical volumetric heating power densities of 8 × 10^11^ W/m^3^ were applied to both integrated heat sources.

### 3.2. Droplet-Induced Thermal Confinement in Three-Phase Flow Regimes

**Figure 5:**
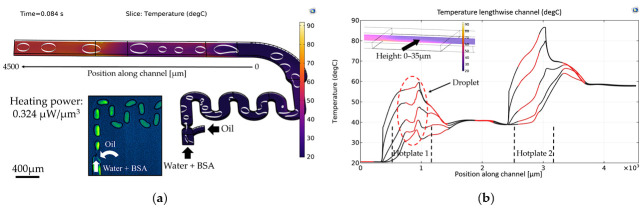
Temperature distribution within generated microdroplets under multiphase flow conditions (**a**). Red segments in the one-dimensional cut-line profiles (**b**) indicate the spatial locations of individual droplets along the channel. Longitudinal profiles show droplets retaining higher temperatures than the carrier phase due to localized heat concentration above the microheaters.

To investigate the effect of droplet presence on the mass transport and thermal behavior within the microfluidic system, the inlet flow rates were adjusted to induce a droplet-generation regime. The total volumetric flow rate was maintained at 1.8 μL/s, with individual inlet flow rates of 0.2 μL/s for water, 0.2 μL/s for the BSA solution, and 1.4 μL/s for the continuous oil phase, resulting in stable droplet formation as illustrated in Figure 5. Owing to the dominant volume fraction and distinct thermal properties of the oil phase, the applied volumetric heating power for both heat sources was reduced to 3.24 × 10^11^ W/m^3^. Data from the longitudinal cut lines and channel mid-plane indicated that droplets experienced substantial thermal accumulation while transiting the dual platinum heating elements. This behavior arises from the comparatively low thermal conductivity of the surrounding oil phase, which effectively acts as a thermal barrier and limits the heat dissipation into the continuous medium.

Thermally confined heating is critical for enabling precise temperature regulation in droplet-based microreactors and biochemical assays. Complementary laboratory experiments employing high-speed imaging confirmed the generation of uniform and regularly spaced droplets under comparable flow conditions, and were further used to assess the differing thermal responses of the constituent liquids.

### 3.3. Droplet Concentration, Homogeneity and Mixing Enhancement

**Figure 6:**
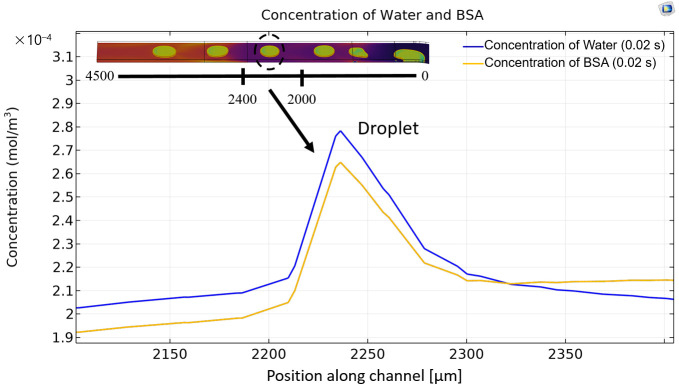
Concentration distribution of mixed water and BSA solutions under multiphase flow conditions. To evaluate the concentration profile, a single longitudinal cut line was defined at the channel mid-plane at a height of 25 µm, using the same spatial extent as in the thermal analysis. The resulting concentration profiles indicate negligible molecular diffusion across the droplet interface, confirming that the dispersed phase remains effectively isolated from the surrounding continuous medium. This confinement allows individual droplets to function as independent microreactors suitable for controlled chemical and biochemical studies.

The concentration distributions of the injected water and BSA solution were further examined under multiphase flow conditions to demonstrate the strong suppression of molecular diffusion across the phase boundaries, along with enhanced intradroplet mixing driven by internal microcirculation. These effects are illustrated in Figure 6.

In curved microchannels, the combined effects of centrifugal forces and laminar Poiseuille flow induce a distortion in the axial velocity profile, shifting the velocity maximum toward the concave wall. This redistribution generates pronounced velocity gradients and an imbalance between the inertial and pressure forces, resulting in the so-called Dean instability. Consequently, secondary flow structures known as Dean vortices emerge, producing transverse circulatory motion across the channel cross-section, thereby enhancing fluid mixing [38]. These secondary flows play a key role in improving mixing efficiency and concentration homogeneity in curved microfluidic geometries. Mixing efficiency (ME) was quantified using the following metric:

(10) $$\mathrm{ME}=(1-\sqrt{\sigma^{2}}/\sqrt{{\sigma_{0}}^{2}})\times 100\%$$

**Figure 7:**
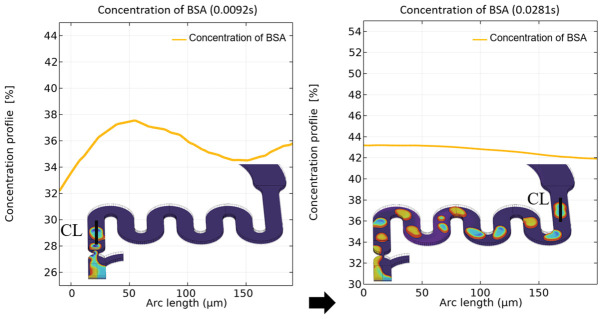
Concentration profiles extracted along a selected cut line (CL) within an individual droplet, showing the spatial distribution of water and BSA solutions at the inlet and outlet of the serpentine mixing channel. A pronounced increase in concentration homogeneity is observed at the downstream location, indicating effective intradroplet mixing.

In (10), σ_0_^2^ denotes the concentration variance under unmixed conditions at the entrance of the serpentine channel, whereas σ^2^ represents the variance evaluated at a given downstream cross section [21]. The evaluation planes were positioned at the midpoints of each curved segment within the serpentine mixing section. Using this approach, the curved microchannels exhibited a high degree of mixing with an average mixing efficiency of 0.72. Such enhanced mixing ensures a uniform distribution of reagents, promotes reaction consistency, and improves the overall reliability of droplet-based microfluidic processes, including biochemical assays and molecular syntheses. To further validate these findings, the local BSA concentration profile within individual droplets was analyzed as they traversed the mixing region of the microfluidic channel. The results are shown in Figure 7.

The lateral concentration distributions at the inlet and outlet of the serpentine section were obtained from the spatial distribution of BSA concentration. The results clearly demonstrate that the combined effects of the channel curvature and droplet internal circulation significantly enhance mixing, leading to a highly homogeneous intradroplet environment. In Figure 6 and Figure 7, the quantitative accuracy of the BSA concentration is limited due to the lower element count (lower mesh resolution), which artificially allows numerical diffusion to occur across the droplet wall interface.

### 3.4. Mesh Convergence Analysis

A mesh convergence study was performed to evaluate the effect of different mesh resolutions on droplet formation, temperature distribution, and concentration profiles of the generated droplets. The mesh types used in this study and their essential parameters are summarized in Table 4.

**Figure 8:**
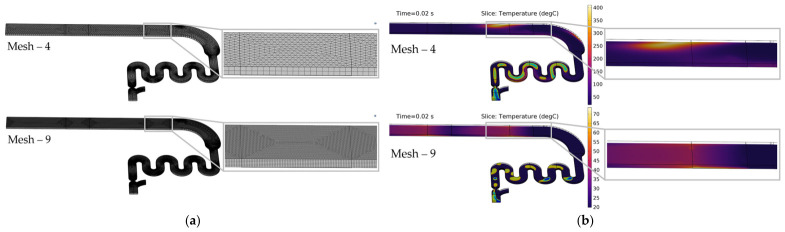
Comparison of mesh resolution effects on droplet formation and temperature field prediction in the microfluidic channel. Computational domains discretized with coarse (Mesh 4) and finer (Mesh 9) mesh resolutions (**a**). Phase distribution of water, BSA, and oil, overlaid with the temperature field at t = 0.02 s, obtained using coarse and finer meshes (**b**). The finer mesh provides improved resolution of the droplet interface and yields a physically consistent temperature distribution confined to the microfluidic domain.

Figure 8 illustrates the influence of mesh resolution on the numerical prediction of droplet formation and the associated thermal field within the microfluidic channel.

**Figure 9:**
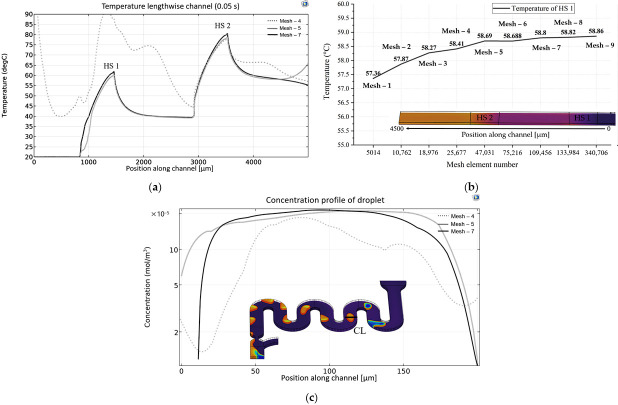
Comparison of mesh resolution effects on temperature and concentration profile predictions. The temperature distribution along the channel length at t = 0.05 s for different mesh types (**a**). Temperature above the first heat source (HS1) measured at a height of 5 µm, illustrating convergence of the predicted temperatures with increasing mesh resolution (**b**) and the concentration profile of water in the first droplet exiting the mixing channel, showing improved interface definition and reduced numerical diffusion as the mesh is improved (**c**).

All mesh configurations (Table 4) were compared based on the temperature and concentration fields. Figure 9a presents the temperature distribution at 0.05 s along the channel cutline, illustrating how the predicted thermal profile evolves with increasing mesh resolution. As the number of elements increased, the temperature gradients near the heat sources became smoother and more consistent, indicating an improved numerical stability. For quantitative comparison, the temperatures above the first heat source (HS1) were evaluated at a height of 5 μm above the channel bottom, as shown in Figure 9b. At lower mesh resolutions, noticeable deviations in the predicted temperatures were observed; however, as the mesh element number increased, the temperature values for HS1 (and HS2) progressively converged, approaching the value of approximately 58.8 °C (and 83.2 °C in case of HS2). Beyond approximately 75,216 elements (Mesh 6), further increasing the mesh element number resulted in only marginal changes in the predicted temperatures, indicating mesh-independent solutions. Mesh 6 was therefore selected for all production simulations. Finally, the effect of mesh resolution on mass transport was examined by analyzing the concentration profile of the first droplet exiting the mixing channel, as shown in Figure 9c. At coarse mesh resolutions, the concentration profile appears diffused and less defined, with smeared interfaces between the phases. Increasing the number of mesh elements leads to sharper concentration gradients and a clearer representation of the droplet structure, demonstrating the improved resolution of interfacial transport phenomena and enhanced numerical accuracy.

### 3.5. Characterization of the Heat Source

**Figure 10:**
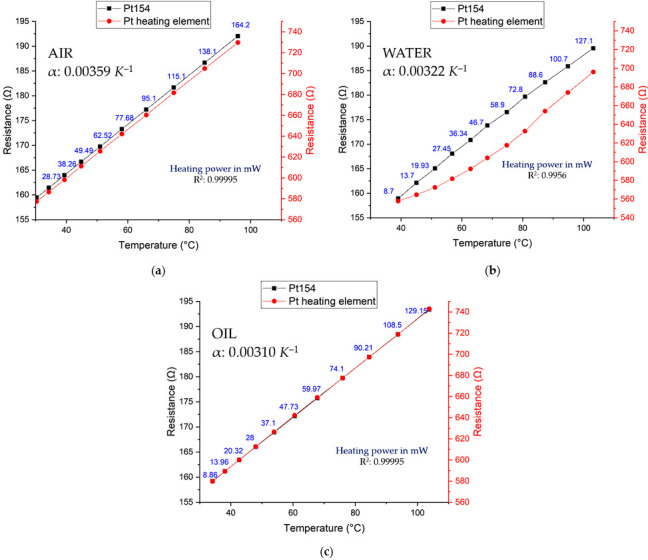
Resistance–temperature characterization of the Pt154 RTD and Pt heating element in an empty channel (**a**), distilled water (**b**), and silicone oil (**c**). The resistance values for both elements increased steadily over the range of approximately 155 to 195 Ω (Pt154 RTD) and 560 to 750 Ω (Pt heating element) in all the three cases.

Electrical measurements were conducted over a temperature range of 20–100 °C for an empty channel and for channels filled with distilled water and silicone oil. Figure 10 shows the measured resistance–temperature relationships for both the platinum resistance temperature detector (RTD) and platinum heating element.

In all three cases (air, water, and oil), the resistance increased linearly with temperature, indicating a stable and repeatable thermal behavior. The total resistance of the temperature-sensing element was approximately 154 Ω, whereas that of the heating element was approximately 540 Ω at room temperature. The extracted temperature coefficients of resistance for the Pt154 RTD were 0.00359 K^−1^ in air, 0.00322 K^−1^ in water, and 0.00310 K^−1^ in silicone oil, closely matching the nominal platinum value of 0.00385 K^−1^. The near-linear response and consistent resistance trends observed across different channel fillings demonstrate that both the sensor and heating element maintain reliable performance under varying thermal boundary conditions. These results confirmed that the integrated heat source can be used for accurate and reproducible temperature measurements and control within the microfluidic system. The slight variations in the extracted temperature coefficients across the different media can be attributed to minor thermal reflections during the infrared camera measurements used to validate the resistance–temperature characterization.

### 3.6. Analyzing the Mixing Phenomenon

**Figure 11:**
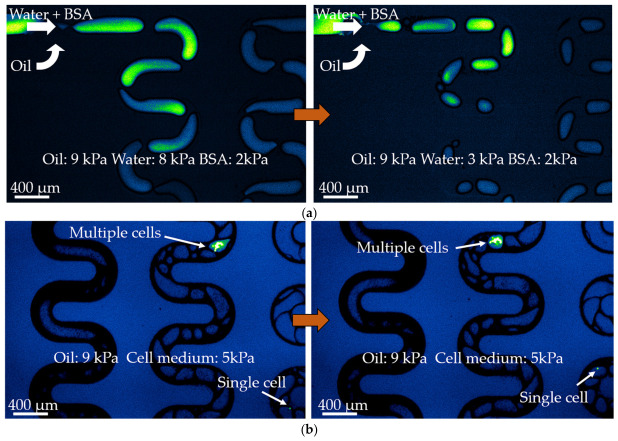
Investigating the Dean vortices inside droplets (**a**). Visualization of internal circulation within droplets flowing through curved microchannels. (**b**) DX5 GFP MES-SA cells inside the droplets exhibit rotational motion along the winding channels due to the centrifugal forces associated with Dean flow.

As illustrated in Figure 11, different pressure settings resulted in distinct droplet morphologies and sizes. The pressure values applied using the CogniFlow micropump produced flow conditions that closely corresponded to the flow rates used in the simulations (expressed in µL/s), resulting in droplets of comparable size. When the droplets reached the first section of the serpentine channel, the oil rind surrounding the droplets underwent torsional effects, causing the droplets to deform, which significantly promoted mixing. Because of this and the centrifugal forces acting on the bends, Dean vortices can be observed within the droplets. To provide additional evidence for this phenomenon, DX5 GFP MES-SA cells were introduced into the droplets, allowing a clearer visualization of how the generated vortices influence the movement and behavior of the cells within the droplets. The pressure conditions applied during these experiments were similar to those used in the initial droplet formation experiments, ensuring comparable flow conditions. Images captured at different positions show droplets containing both single and multiple cells, confirming that Dean-induced internal circulation effectively homogenizes the biological content prior to thermal treatment, which is a critical prerequisite for reproducible heat-shock experiments. The pressure-dependent droplet morphology data in Figure 11a establish the operating window for droplet generation and confirm agreement between the experimental flow conditions and simulation parameters, while Figure 11b demonstrates the platform’s capability to handle and manipulate live cancer cells within the droplets. Together, these results provide direct experimental evidence of Dean vortex-driven intradroplet mixing. Within this homogenized environment, the primary focus is on isolating single cells to eliminate population averaging and paracrine signaling, enabling precise mapping of independent cellular survival thresholds. However, because the system naturally generates both single- and multi-cell droplets, it provides a built-in comparative framework to study solitary resistance alongside cooperative cellular defense responses under identical stress.

While cell occupancy per droplet was assessed through fluorescence microscopy inspection of representative droplets in this proof-of-concept study, comprehensive droplet-by-droplet cell counting was not performed; this is acknowledged as a current limitation, and automated inline cell number verification is identified as a key quality control improvement for future iterations of the platform.

### 3.7. Thermal Treatment and Fluorescence Loss Under Controlled Heating

**Figure 12:**
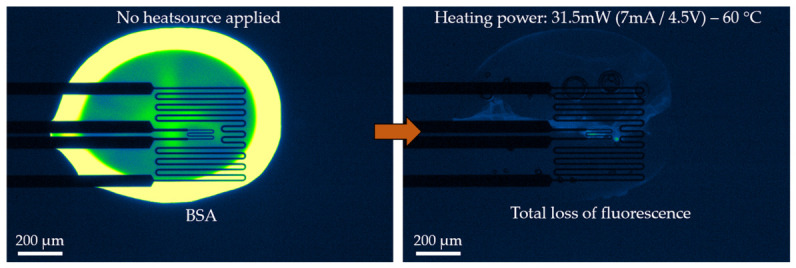
Fluorescent BSA droplet before and after localized thermal exposure. The left image shows a bright, uniformly fluorescent BSA droplet in the absence of heating, while the right image illustrates the near-complete loss of fluorescence following application of a heating power of 31.5 mW (7 mA, 4.5 V) reaching a temperature value around 60 °C. The rapid fluorescence decay indicates thermally induced protein denaturation, demonstrating the effectiveness of the integrated micro-heater in locally disrupting protein stability.

**Figure 13:**
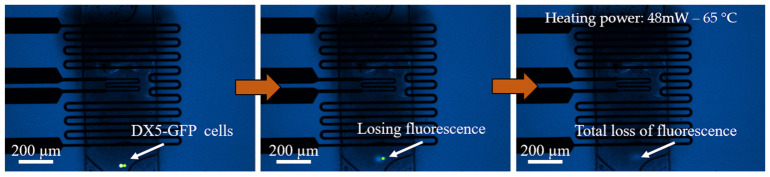
Progressive fluorescence loss of DX5-GFP cancer cells during localized heating. Sequential frames show individual GFP-expressing cells encapsulated in microdroplets as they pass near the integrated micro-heater. Increasing thermal exposure leads to gradual fluorescence attenuation, culminating in complete signal extinction, which is indicative of irreversible protein denaturation and cell death at 62–65 °C. This response reflects the breakdown of cellular thermoprotection mechanisms, including heat shock protein-mediated pathways, under controlled heat-shock conditions.

To characterize the thermal response of biomolecules and cancer cells under localized heating, fluorescently labeled biomolecules and cancer cells were subjected to controlled heating using an integrated platinum microheater. Specifically, BSA solution (Figure 12) and DX5-GFP cancer cells (Figure 13) were investigated as model systems to identify the temperature and electrical power thresholds at which fluorescence loss occurs due to protein denaturation or irreversible cellular damage. This study focuses primarily on the monitoring of controlled thermal heating; within this context, the observed 60–65 °C range for total fluorescence loss serves as a robust indicator of thermal stress sufficient to compromise protein integrity. While this temperature range results in non-specific thermal killing rather than the selective HSP-mediated sensitization typical of canonical heat-shock ranges (41–43 °C) [12,13,14,15], determining these thresholds is essential for defining operational regimes that effectively sensitize cells within microfluidic platforms. Consequently, while the current work establishes the parameters for thermal control, the specific investigation of HSP deactivation and its downstream impact on drug resistance is identified as the primary objective of future biological validation work.

In the first experiment, a single BSA droplet was positioned directly on the heater surface without the PDMS microchannel (Figure 12). Upon application of a heating power of 31.5 mW (7 mA, 4.5 V) and the reached approximately 60 °C surface temperature, the fluorescent signal completely decayed within a few seconds. This rapid fluorescence loss indicated thermally induced protein unfolding and hydrolysis, confirming that the applied heat was sufficient to disrupt the protein structure. The BSA solution used in these experiments was prepared at a concentration of approximately 10 mg/mL, which is physiologically relevant to total intracellular protein concentrations in mammalian cells [39]. Also, the agreement between the BSA denaturation threshold and the cell death threshold supports the use of BSA as a reliable functional proxy for validating the thermal performance of the platform before introducing live cells. Because BSA serves as a surrogate for intracellular proteins, these results establish a baseline for the thermal conditions required to overcome protein stability and chaperone-mediated protection mechanisms.

Subsequently, DX5-GFP cancer cells encapsulated within the microdroplets were examined under more representative microfluidic conditions (Figure 13). In this configuration, the PDMS microchannel is bonded to the heater and filled with either water or silicone oil. While stable droplet generation was achieved, the high flow velocities within the serpentine channel limited the precise temporal tracking of the fluorescence decay. Nevertheless, at elevated heating powers, GFP fluorescence consistently vanished within seconds, indicating severe thermal stress leading to protein denaturation and cell death. In rare instances, where cells remained stationary near the heater, a more accurate determination of the lethal thermal threshold was possible. Under zero-flow conditions, complete fluorescence loss, corresponding to irreversible cellular damage was observed at a minimum heating power of 48 mW (9 mA, 5.3 V).

The flow rates used in the FEM simulations and laboratory experiments (0.2–1.4 µL/s per inlet) and associated thermal power levels (31.5–48 mW) are consistent with the low-power operation characteristic of integrated resistive microheater platforms reported in comparable microfluidic hyperthermia and on-chip PCR studies [22,23]. In clinical hyperthermia, tumor tissue is typically heated to 40–45 °C and maintained at this temperature for 30–60 min using radiofrequency or focused ultrasound delivery systems, at power densities substantially exceeding those employed in microfluidic platforms [40,41,42], whereas the present platform applies short, localized heat pulses at the single-droplet level. Regarding total energy absorbed: with a droplet passage time above the heater of 1–3 s, as directly measured from the fluorescence microscopy recordings, the delivered energy per droplet is 48–144 mJ at 61 mW. Droplets of 100–150 µm width in the 50 µm deep channel have an estimated volume of 0.4–1.5 nL, corresponding to a volumetric energy density of approximately 32–360 kJ/mL. While this substantially exceeds clinical dose levels, the energy is applied transiently and locally, consistent with the intended use as an acute in vitro thermal stress model rather than a sustained therapeutic modality. Nevertheless, the precise and controllable nature of on-chip thermal delivery demonstrated here offers a potential pathway toward personalised pre-clinical screening of patient-derived cancer cells, where individual thermal sensitivity profiles could inform the optimisation of clinical hyperthermia protocols.

Some limitations of the current study should be acknowledged. The thermal characterisation relies on fluorescence loss as a proxy for cell death, which, while well-established, does not directly measure intracellular HSP activity or downstream drug sensitisation; dedicated biochemical assays will be required in future work to validate HSP-mediated mechanisms. Cell loading uniformity was verified by manual fluorescence inspection rather than automated counting, introducing variability in the cell-per-droplet statistics. The experiments were conducted under static or low-flow conditions for the most precise thermal threshold determination, whereas dynamic flow conditions introduce variability in droplet residence time above the heater; a flow-synchronised heating strategy or droplet trapping mechanism would improve reproducibility.

## 4. Conclusions

This work presented a droplet-based microfluidic platform for controlled local thermal treatment of encapsulated cancer cells, supported by multimodal numerical simulations and experimental validation. The simulations captured the coupled behavior of fluid flow, heat transfer, and species transport within droplets, enabling the detailed prediction of temperature distributions and concentration profiles under localized heating conditions. The numerical results demonstrated that the proposed design provided well-controlled and spatially localized heating together with enhanced mixing inside the droplets. Importantly, the simulated temperature fields and concentration trends showed good agreement with laboratory measurements, confirming the accuracy and reliability of the modeling approach. This agreement supports the use of simulations as a predictive tool for optimizing heater placement, operating conditions, and droplet residence time. Overall, the combined experimental–numerical approach established a robust framework for the development of microfluidic systems capable of precise thermal and chemical manipulation at the single-droplet level. Such control is highly relevant for microscale hyperthermia, drug delivery, and cell-based assays and provides a foundation for future biological studies involving targeted enhanced chemotherapeutic cancer treatment. Building upon this established engineering framework, future research will focus on enhancing the platform’s operational robustness and expanding its application in combined thermal and chemical stimulation. To facilitate experiments in higher voltage regimes, the integrated heat source will be modified to suppress electrochemical hydrolysis and ensure long-term heating stability. These technical refinements will enable a more systematic evaluation of cellular responses to varying concentrations of chemical stimuli, such as hydrogen peroxide, within the homogenized intradroplet environment. Furthermore, we aim to investigate the synergistic effects of stable thermal stimuli combined with clinically relevant therapeutic agents. By precisely quantifying the interplay between localized heat shock and drug efficacy, this approach seeks to provide a scalable strategy for overcoming multidrug resistance in aggressive cancer lineages.

Despite the abovementioned limitations, the present platform represents a meaningful step toward a fully integrated, controllable microfluidic tool for thermal manipulation cancer cells, with direct relevance to advanced cancer therapy research. Its modular design—combining FEM-guided fabrication, on-chip Pt heating, and Dean vortex mixing—provides a flexible foundation for combined chemo-thermal sensitisation studies, single-cell drug resistance profiling, and high-throughput screening of therapeutic heat doses in patient-derived tumor cell lines, all of which are identified as key directions for future work.

## Figures and Tables

**Table 1:** Material properties for flow characteristics.

Material	Dynamic Viscosity (μ)	Density (ρ)
Distilled water	0.89 × 10^−3^ Pa·s	10^3^ kg/m^3^
Silicone Oil	20 × 10^−3^ Pa·s	10^3^ kg/m^3^
BSA Solution	10^−3^ Pa·s	1.03 × 10^3^ kg/m^3^
Platinum	—	21.5 × 10^3^ kg/m^3^

**Table 2:** Transport properties for mixing.

Material	Concentration (C_i_)	Diffusion Coefficient (Dc_i_)
Distilled Water	55 mol/m^3^	2.3 × 10^−9^ m^2^/s
Silicone Oil	10 mol/m^3^	0.5 × 10^−10^ m^2^/s
BSA Solution	1.51 × 10^−4^ mol/m^3^	6.7 × 10^−11^ m^2^/s

**Table 3:** Thermal properties.

Material	Heat Capacity (C_p_)	Ratio of Specific Heats (Γ)	Thermal Conductivity at 20 °C (κ)
Distilled water	4182 J/(kg·K)	1	0.6 W/(m·K)
Silicone Oil	2000 J/(kg·K)	0.17	0.16 W/(m·K)
BSA Solution	4100 J/(kg·K)	1	0.6 W/(m·K)
Platinum	125.6 J/(kg·K)	0.036	71.6 W/(m·K)

**Table 4:** Mesh properties.

Mesh	Elements	Average Element Quality	Min. Size (μm)	Max. Size (μm)
1	5014	0.9114	12.1	48.4
2	10,762	0.9307	9.67	31.4
3	18,976	0.8995	14.0	52.0
4	25,677	0.9458	7.25	24.2
5	47,031	0.9589	4.84	16.2
6	75,216	0.9327	13.0	34.0
7	109,456	0.9654	2.51	13.2
8	133,984	0.9402	8.20	25.0
9	340,706	0.9793	0.97	8.95

## Data Availability

The data are contained within the article.

## Abbreviations

The following abbreviations are used in this manuscript:

FEM	Finite Element Method
BSA	Bovine Serum Albumin
MES-SA	Uterine sarcoma cell line
PCR	Polymerase Chain Reaction
HSP	Heat Shock Protein
CFD	Computational Fluid Dynamics
PDMS	Polydimethylsiloxane
PCB	Printed Circuit Board
SMU	Source Measure Unit
RTD	Resistance Temperature Detector
GFP	Green Fluorescent Protein
